# Cost-Benefit Analysis of Enzymatic Hydrolysis Alternatives for Food Waste Management

**DOI:** 10.3390/foods14030488

**Published:** 2025-02-03

**Authors:** Cristina Ghinea, Elena-Diana Ungureanu-Comăniță, Raluca Maria Țâbuleac, Paula Sânziana Oprea, Ersilia Daniela Coșbuc, Maria Gavrilescu

**Affiliations:** 1 Faculty of Food Engineering, Stefan cel Mare University of Suceava, 720229 Suceava, Romania; cristina.ghinea@fia.usv.ro; 2 Department of Environmental Engineering and Management, “Cristofor Simionescu” Faculty of Chemical Engineering and Environmental Protection, “Gheorghe Asachi” Technical University of Iasi, 73 Prof. D. Mangeron Blvd., 700050 Iasi, Romania; raluca-maria.tabuleac@student.tuiasi.ro (R.M.Ț.); paula-sanziana.oprea@student.tuiasi.ro (P.S.O.); ersilia-daniela.cosbuc@student.tuiasi.ro (E.D.C.); 3 Academy of Romanian Scientists, 3 Ilfov Street, 050044 Bucharest, Romania; 4 Academy of Technical Sciences of Romania, 26 Dacia Blvd., 030167 Bucharest, Romania

**Keywords:** bioethanol, bioactive peptides, enzymatic hydrolysis, food waste, nutrients, organic acids, waste management

## Abstract

In this study, we aimed to evaluate the economic, environmental, and social viability of the enzymatic hydrolysis process for food waste valorization by applying cost-benefit analysi (CBA). Our research was based on the investigation of three scenarios/alternatives for the final product of the enzymatic hydrolysis process and the production of bioethanol, bioactive peptides, and organic acids. Key economic indicators, such as cost/benefit ratios, net present value (NPV), and internal rate of return (IRR), were used to evaluate financial performance. At the end of the CBA, a sensitivity analysis was conducted to highlight the performance of each scenario under varying conditions, including fluctuating costs, benefits, and discount rates. These results indicate that enzymatic hydrolysis offers a significant opportunity for reducing food waste and its environmental impacts and promotes sustainability. Bioactive peptide production was found to be the most environmentally viable option, offering the highest cost-benefit efficiency. In both the optimistic and pessimistic scenarios of the sensitivity analysis, the results revealed that bioactive peptide production is economically viable, while the other alternatives, such as bioethanol and organic acid production, are more sensitive to economic and operational changes. This study revealed that enzymatic hydrolysis, as evaluated through CBA, offers a viable and impactful method for food waste management. It reduces environmental impacts, enhances sustainability, and aligns with the principles of a circular economy. The adoption of such innovative waste management strategies is considered essential for building a more sustainable and resource-efficient food system.

## 1. Introduction

The global generation of waste has become a critical environmental issue, posing significant risks to ecosystems and human well-being, with food waste representing a considerable share. Key drivers of this challenge include rapid urbanization, population growth, and unsustainable consumption patterns, which have intensified the pressure on waste management systems. Presently, approximately 2.01 billion tons of municipal waste are generated annually, with at least 33% inadequately recovered. This insufficient waste management contributes to greenhouse gas emissions, soil and water pollution, and biodiversity loss. Projections suggest that by 2050, global waste production will increase by 70%, reaching approximately 3.40 billion tons, exceeding the rate of population growth [1,2,3]. These trends underscore the urgent need for sustainable waste management strategies aimed at mitigating environmental impacts and minimizing adverse socioeconomic effects.

### 1.1. Sources, Impacts, and Management Strategies of Food Waste

Food waste represents a significant fraction of the total municipal solid waste, with its proportion varying across regions depending on social, economic, and consumption patterns. In high-income countries, food and organic waste account for approximately 32% of the total waste. In contrast, in low- and middle-income countries, this proportion increases to 53–57%, reflecting greater reliance on fresh food and less efficient food resource management systems [2,3]. These disparities highlight the influence of socioeconomic factors on waste composition and management practices.

**Figure 1:**
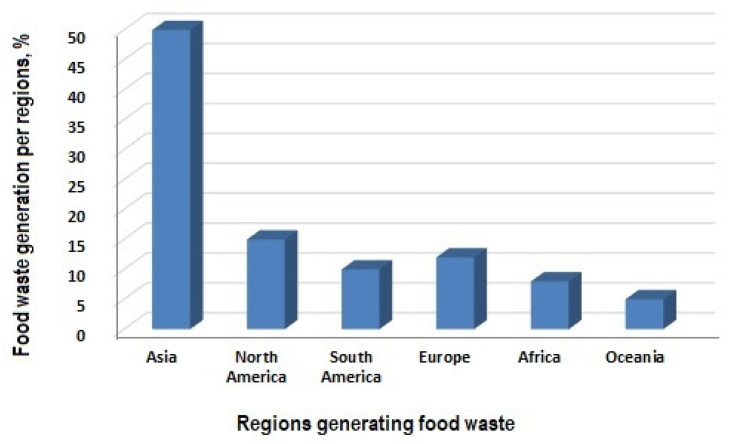
The approximate percentage of global food waste generation by region (drawn by authors following data from FAO, https://www.fao.org/4/mb060e/mb060e02.pdf (accessed on 25 November 2024), https://openknowledge.fao.org/server/api/core/bitstreams/0c372c04-8b29-4093-bba6-8674b1d237c7/content (accessed on 25 November 2024).

**Figure 2:**
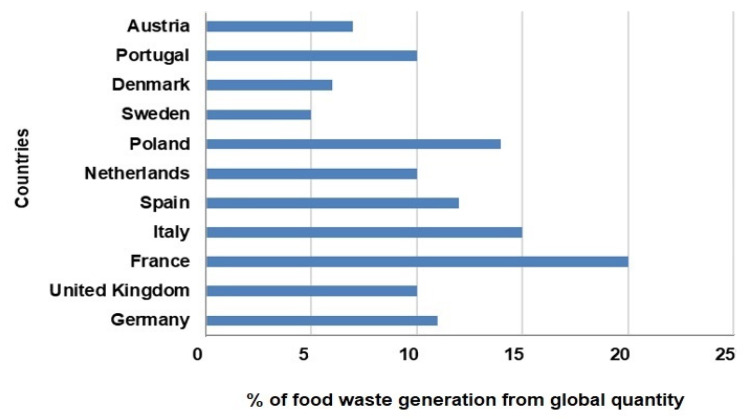
The approximate percentage of global food waste generation in Europa by countries (drawn by authors following data from FAO, https://www.fao.org/4/mb060e/mb060e02.pdf (accessed on 25 November 2024), https://openknowledge.fao.org/server/api/core/bitstreams/0c372c04-8b29-4093-bba6-8674b1d237c7/content (accessed on 25 November 2024)).

Globally, Asia stands out as the largest contributor to food waste generation, followed by North America and Europe. Smaller yet noteworthy contributions come from South America, Africa, and Oceania, as depicted in Figure 1. In Europe, food waste levels also vary significantly. As shown in Figure 2, France exhibits the highest food waste generation rate, exceeding 20%, whereas countries such as Austria and Sweden demonstrate much lower contributions, ranging between 5% and 7% [3]. These regional differences underscore the importance of tailoring waste management strategies to effectively address specific consumption behaviors and economic contexts.

Food waste is generated across various stages of the food supply chain, from production to consumption, and encompasses both food loss and waste. Key sources of food waste include the following [4,5]:

(a) Agricultural production

Losses occur during harvesting due to inefficient techniques, adverse weather conditions, or crops being rejected based on market or buyer quality standards.

(b) Processing

Significant food waste arises from incorrect processing methods, prolonged storage, products exceeding expiry dates, and packaging defects.

(c) Distribution and trade

Waste at this stage is often due to transportation issues, mishandling, or inadequate storage conditions, such as improper temperature or humidity control.

(d) Final consumers

At the household and restaurant levels, food waste is predominantly caused by over-purchasing, improper storage practices, and misunderstandings about expiry dates [4,6].

Table 1 presents the percentage of food waste by country, detailing sector-specific waste breakdowns in various European nations. For instance, Italy and Spain report substantial food waste at the household level, accounting for 65% and 60% of the total food waste, respectively. These figures suggest the influence of cultural habits, consumer behavior, and logistical inefficiencies on food waste challenges [4,6]. Understanding these sources and their relative contributions is essential for designing effective strategies to minimize food waste at each stage of the supply chain.

According to the Food and Agriculture Organization of the United Nations (FAO), nearly one-third of the food produced worldwide, representing approximately 1.3 billion tons annually, is lost or wasted [1,4]. This enormous volume highlights inefficiencies in the global food system, with significant repercussions for the environment, economy, and society. Ineffective food waste management not only results in the loss of valuable resources but also contributes heavily to methane emissions from landfills, a potent greenhouse gas that accelerates climate change [1,5].

Globally, food loss and waste produce about 8% of all human-made greenhouse gas emissions, with a carbon impact similar to that of some of the world’s largest polluting countries [4]. For every kilogram of food produced, an estimated 4.5 kg of CO_2_ is released into the atmosphere, emphasizing the considerable environmental burden associated with food waste [6]. Beyond its carbon impact, food waste depletes essential resources, such as soil, water, and energy, throughout the lifecycle of food products, exacerbating resource scarcity and environmental degradation [4].

The implications of food waste extend beyond environmental concerns and carry profound ethical and social dimensions. While millions of tons of food are discarded annually, approximately 793 million people around the world face chronic malnutrition [7]. This stark disparity underscores the urgent need to rethink global food distribution and waste management systems to address hunger, improve resource efficiency, and promote sustainability [1,7]. Addressing food waste is not merely an environmental and economic priority, but also a moral imperative, reflecting the interconnected challenges of equity, sustainability, and global food security.

**Table 1:** Food waste by sector in European countries *.

Country	Household (%)	Retail (%)	Production (%)	Food Service (%)	Total Food Waste (%)
Germany	60	10	20	10	~11
UK	50	15	20	15	~10
France	55	15	20	10	~20
Italy	65	10	15	10	~15
Spain	60	12	15	13	~12
The Netherlands	50	20	20	10	~10
Poland	55	15	20	10	~14
Sweden	70	10	10	10	~5
Denmark	65	15	10	10	~6
Portugal	60	15	15	10	~10
Austria	60	15	15	10	~7
Romania	60	15	15	10	~13

* Elaborated by authors based on Eurostat data (https://ec.europa.eu/eurostat/statistics-explained/index.php?title=Food_waste_and_food_waste_prevention_-_estimates&stable=0&redirect=no (accessed on 29 November 2024)).

### 1.2. Impact of Food Waste Generation

The environmental impact of food waste is multifaceted, encompassing a wide range of detrimental effects on the environment, such as:

- Greenhouse Gas (GHG) emissions

When food waste decomposes in landfills under anaerobic conditions, it produces methane, a greenhouse gas that is 28–34 times more potent than carbon dioxide in the long term and more than 80 times more potent in the short term [8,9].

- Wastage of natural resources

Food production consumes vast amounts of natural resources. For instance, producing just one kilogram of beef requires approximately 15,000 L of water. When food is wasted, water, energy, and other resources used in its production are also squandered [9,10].

- Soil and water pollution

Improperly managed food waste in landfills generates leachate, a toxic liquid containing organic and inorganic substances, including heavy metals and harmful compounds, which can contaminate soil and groundwater, posing serious risks to local ecosystems and human health [8,11].

- Biodiversity loss

The demand for additional agricultural land to produce food that may go to waste drives deforestation, destruction of natural habitats, and reduced biodiversity, negatively affecting wildlife populations [9,12].

- Air pollution

In addition to methane, the decomposition of food waste emits volatile organic compounds (VOCs) and other toxic gases that contribute to air quality degradation, smog formation, and broader public health issues [9,10].

- Contribution to climate change

The combined direct and indirect GHG emissions from food waste account for 8–10% of the annual global emissions, surpassing those of the entire global aviation sector [10,12].

Food waste significantly impacts human health and welfare through pollution, attraction of disease vectors, and inefficiencies in resource use. These impacts manifest in the following direct and indirect ways:

- Air quality and respiratory diseases
  - During the decomposition of food waste, harmful gases, such as methane and volatile organic compounds (VOCs), are released, degrading air quality; however, exposure to these emissions can cause respiratory irritation, asthma, and other lung diseases [10,13].
- Water and soil contamination
  - The decomposition of food waste in landfills generates leachate, a toxic liquid containing heavy metals, pathogenic bacteria, and other harmful substances that can contaminate groundwater, leading to severe gastrointestinal illnesses among populations consuming the affected water [11].
  - If improperly treated, the food waste used in compost can introduce harmful bacteria into the food chain, posing risks of contamination and endangering public health [12].
- Disease vectors
  - Food waste that is not adequately managed attracts rodents, insects, and other pests, which act as vectors for infectious diseases like leptospirosis, salmonellosis, and malaria. Such diseases are particularly prevalent in urban areas with inadequate waste management systems [13].
  - Wasted food often serves as a breeding ground for pathogenic bacteria, such as *Escherichia coli* and *Listeria monocytogenes*, which are common causes of serious foodborne illnesses [8,13].
- Economic and social impacts
  - Food waste represents the loss of valuable agricultural, economic, and natural resources that could otherwise be used to alleviate food insecurity. This inefficiency exacerbates malnutrition, especially in developing regions where access to food is already limited [10].
  - Communities often bear the economic burden of managing food waste through the costs associated with pollution, disease treatment, and lost productivity caused by poor health outcomes [10,12].
- Long-term consequences
  - Inadequate food waste management can contribute to the spread of antimicrobial resistance by introducing resistant bacteria into the environment. This global health threat poses severe challenges to treating infectious diseases effectively [11,12].

### 1.3. Sustainability of Food Waste Management at National and International Levels

Transitioning to a circular economy in food waste management represents a vital step toward achieving a sustainable economic system in which resources are reused, recovered, and reintegrated into production processes to their full potential. In this model, food waste is no longer regarded as waste but as a valuable resource that can be transformed into useful products, such as bioenergy, compost, or raw materials for industrial applications [14,15].

Urgent measures are required to address the persistent challenges of food waste, including enhanced waste collection systems and the promotion of circular economy principles. The recycling and recovery of natural and energy resources play a central role in this transition, enabling the transformation of food waste into a source of economic and environmental value [16,17]. At both the national and international levels, food waste management encompasses a range of strategies and policies aimed at reducing waste generation and mitigating its environmental impacts. These efforts are supported by legislation, government initiatives, and international collaborations that promote sustainable practices, foster innovation, and encourage the adoption of waste recovery technologies [18,19].

The shift toward a circular economy in food waste management not only addresses the environmental and resource challenges associated with waste but also aligns with broader goals of sustainability, resilience, and economic efficiency. Such systemic changes are critical for building a sustainable future that optimizes resource use while minimizing environmental degradation.

#### 1.3.1. National Actions

Efforts to combat food waste at the national level include a variety of strategies, programs, and collaborations aimed at reducing waste generation, promoting resource recovery, and raising public awareness.

- Programs to reduce food waste
  - National strategies often focus on creating frameworks to minimize food waste. For instance, Romania’s Law 217/2016 encourages the redistribution of near-expiry food to charities, ensuring that surplus food is utilized rather than discarded.
  - Tax incentives and similar economic measures are also employed to encourage businesses to adopt sustainable practices and reduce waste.
- Public awareness campaigns
  - Educational initiatives target households, encouraging them to adopt waste-reducing habits, such as planning grocery shopping, composting food scraps, and separating organic waste. These campaigns aim to foster behavioral change and improve waste management at the individual level [20,21].
- Recycling and composting programs
  - The separate collection of organic waste is a key focus of many national programs, facilitating its transformation into compost or biogas. For example, in Romania, several municipalities have implemented selective organic waste collection programs that enable effective recycling and energy recovery [22,23].
- Public-private partnerships
  - Collaboration between government entities, private companies, and non-governmental organizations (NGOs) has been instrumental in advancing technological solutions for food waste valorization. These partnerships support innovative uses of food waste, such as its conversion into bioenergy or other valuable byproducts, driving sustainability and economic efficiency [24].

These actions illustrate the multifaceted approaches taken at the national level to tackle food waste, combining legislative measures, community engagement, and technological advancements to reduce waste, recover resources, and minimize environmental impact. Such initiatives play a critical role in fostering sustainable food systems and achieving broader environmental and economic goals.

#### 1.3.2. International Actions

Efforts to address food waste on a global scale are driven by collaborative international actions, policies, and technological advancements aimed at promoting sustainability and resource efficiency. Key initiatives include the following:

- Sustainable Development Goals (SDGs)
  - The United Nations Agenda for Sustainable Development includes Goal 12, which aims to ensure sustainable consumption and production patterns. Specifically, Target 12.3 seeks to halve global food waste per capita by 2030 at both the retail and consumer levels, addressing food losses along the production and supply chains [25].
- Food and Agriculture Organization (FAO)
  - The FAO actively supports member states in the development and implementation of food waste reduction policies. These efforts include providing technical expertise, fostering policy dialogue, and facilitating global partnerships to strengthen food systems and reduce waste [26].
- Regional initiatives
  - The European Union (EU) has integrated food waste reduction into its broader environmental strategies, such as the European Green Deal and the Circular Economy Action Plan. These policies establish ambitious targets for food waste reduction across all member states and encourage innovative practices to optimize resource use while minimizing waste [27,28].
- Food recovery programs
  - In the United States, programs like the “US Food Loss and Waste 2030 Champions” initiative encourage businesses to commit to cutting their food waste by 50% by 2030. Such initiatives foster private sector engagement in achieving national and global food waste reduction goals [29].
- Technological innovations and research
  - Advances in technology and scientific research have introduced innovative methods for recovering and repurposing food waste [30,31].

## 2. Innovative Solutions and Technologies to Reduce Food Waste

Innovative technologies play an important role in the effective management of food waste. The following are some emerging solutions and technologies for reducing food waste (Table 2). Enzymatic hydrolysis uses enzymes to break down food waste into simpler substances, such as bioethanol, organic acids, and bioactive peptides. This is an efficient process that transforms waste into a source of renewable energy and other valuable products [32,33]. Advanced composting technology uses microorganisms and controlled conditions to accelerate the process and obtain high-quality compost faster. Modern technologies include controlled aerobic composting and anaerobic digestion [34,35]. Food waste bioreactors use bacteria and microorganisms to break down food waste and produce biogas (methane and carbon dioxide). They can be used to generate electricity or heat [36]. Food preservation and processing technologies for extending shelf-life packaging and processing technologies, such as smart packaging and freeze-drying or freeze-drying, can help extend the shelf life of food products and reduce food waste [37,38].

AI and big data for supply chain optimization can be used to analyze food flows in supply chains and anticipate consumer demand [39,40]. Digital food redistribution platforms, such as mobile applications that connect restaurants, stores, and consumers to redistribute unused food to charities, are effective solutions for combating food waste [41,42].

Ferments and fermented foods use microbial cultures to ferment expired or uneaten food. Fermented foods can be used to create new products, such as probiotic beverages or nutritional ingredients [43,44]. These technologies not only contribute to environmental protection but also promote a more equitable and resource-efficient food system. Their implementation can transform food waste management into an economic and ecological opportunity [45].

Various studies in the literature have also investigated innovative solutions and technologies to reduce food waste, demonstrating different technological approaches. Some studies have been conducted to explore the use of enzymatic hydrolysis for converting food waste into bioethanol. They showed that by using specific enzymes, organic waste can be broken down into compounds that can be fermented to produce bioenergy, which contributes to the reduction of food waste and renewable energy generation [46,47]. In addition, the potential of food waste valorization was studied using enzymatic hydrolysis to produce organic acids and bioethanol. It was concluded that this technology can efficiently transform waste into value-added products, while providing an environmentally friendly solution for food waste management [48,49].

Some researchers have discussed the use of composting and anaerobic digestion for organic waste management in the food industry, showing that these methods not only reduce waste volumes but also contribute to cost savings and methane emission reductions [50,51]. In addition, some studies analyzed the impact of mobile apps that would facilitate food donations from consumers to charitable organizations, showing that these solutions contribute significantly to reducing food waste and helping people in need [52,53]. The use of smart packaging technologies has been found to extend the shelf life of perishable foods such as fruits and vegetables. Smart packaging can monitor and control storage conditions, such as temperature and humidity, to prevent rapid food degradation and reduce food waste [54,55].

## 3. Transforming Food Waste into Valuable Products by Enzymatic Processes

The transformation of food waste into valuable products represents a cornerstone of sustainable waste management strategies aligned with the principles of the circular economy [12,15]. By converting waste into high-value materials, industries can mitigate environmental damage while generating economic benefits, thus fostering a more resource-efficient and environmentally friendly system.

Food waste, often perceived as a liability, is rich in organic compounds that can serve as feedstock for a variety of industrial processes. These compounds include carbohydrates, proteins, and lipids, which are readily convertible into biofuels, biochemicals, and functional materials. Exploiting these resources not only reduces the volume of waste sent to landfills but also creates opportunities for innovation across sectors.

**Figure 3:**
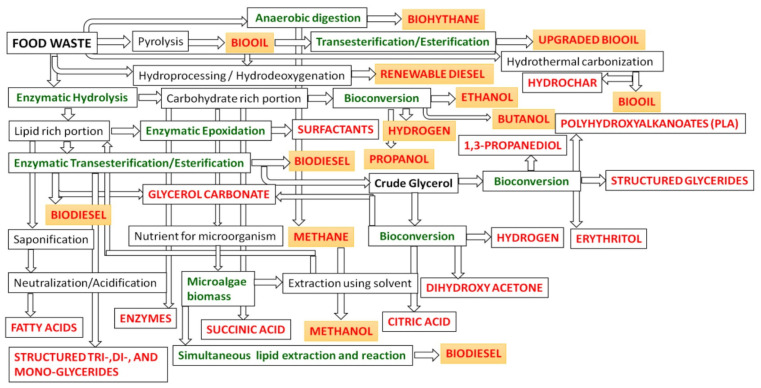
A proposed food waste-based biorefinery using biocatalysis (green color shows the methods can be performed using enzymes, marked in orange: gaseous biofuels: hydrogen, methane, and biohythane; liquid biofuels: biodiesel, methanol, ethanol, propanol, butanol, bio-oil, upgraded bio-oil, and renewable diesel) (reused from Karmee, 2023 [33], under the terms and conditions of the Creative Commons Attribution (CC BY) license (https://creativecommons.org/licenses/by/4.0/ (accessed on 29 November 2024)).

Enzymatic hydrolysis is a key step in transforming food waste into valuable products by employing targeted enzymes to break down complex biomolecules into simple, high-value compounds. Various enzymatic processes, including hydrolysis, esterification, transesterification, interesterification, oxidation, reduction, decarboxylation, anaerobic digestion, fermentation, and epoxidation, can be applied effectively for waste valorization. In this context, anaerobic digestion (biogasification) is used to transform food waste into biohythane and methane (Figure 3). The methane produced can be enzymatically converted to methanol through the action of methane mono-oxygenases (MMOs). Thermochemical conversion methods are utilized to generate bio-oil from food waste, which is then enhanced to a higher-quality fuel with the help of lipase enzymes (Figure 3). Additionally, bioconversion techniques have been employed to process the lipid content of food waste into biodiesel, while the carbohydrate-rich fraction is directed toward ethanol production. The carbohydrate portion can also serve as a nutrient source for cultivating microalgae and producing enzymes. The resulting algae biomass undergoes simultaneous lipid extraction and enzymatic transesterification to produce biodiesel (Figure 3). Moreover, other biofuels such as propanol and butanol are also synthesized through biocatalytic processes [33].

**Figure 4:**
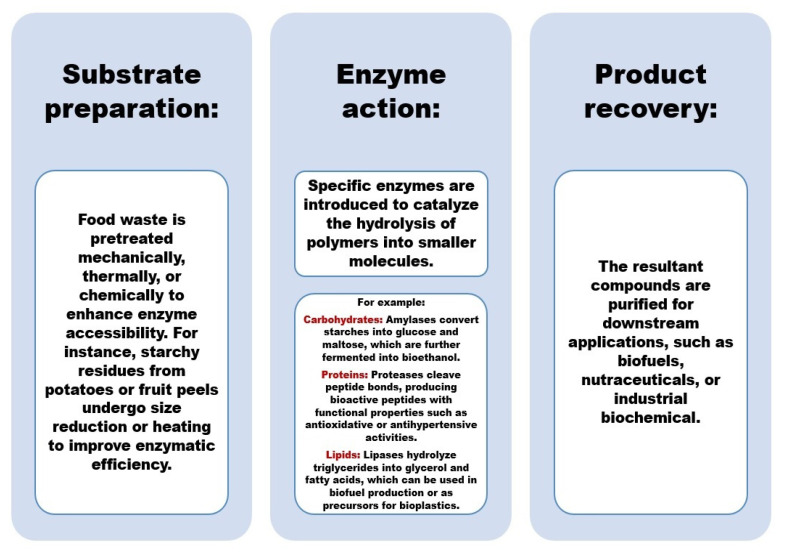
Steps of the enzymatic hydrolysis process.

The enzymatic process utilizes the catalytic action of specific hydrolytic enzymes, such as amylases, proteases, and lipases, which target and break down carbohydrates, proteins, and lipids, respectively, into simpler and more usable components. Amylases catalyze the degradation of complex carbohydrates into simple sugars, proteases break down proteins into peptides and amino acids, while lipases hydrolyze lipids into glycerol and free fatty acids. This process involves three main stages, which are outlined in Figure 4. Initially, enzymes bind to their respective substrates through specific interactions at the active site, forming an enzyme-substrate complex. The second stage involves a catalytic reaction, where the chemical bonds in the substrate are cleaved, leading to the formation of simpler molecules. In the final stage, the reaction products are released, and the enzyme remains available to catalyze further reactions.

As illustrated in Figure 4, these sequential steps are critical for efficient enzymatic hydrolysis, making it an essential mechanism for industrial processes such as biofuel production, food waste valorization, and biopolymer degradation.

One of the most prominent products derived from food waste is bioethanol, a renewable energy source that has gained considerable attention because of its role in reducing reliance on fossil fuels and lowering greenhouse gas emissions (Table 3). Bioethanol is produced through the fermentation of carbohydrate-rich food waste, such as starchy residues and fruit peels, using microbial processes. The resulting bioethanol can be used as a clean fuel alternative for transportation and energy generation, contributing to global efforts to combat climate change.

**Figure 5:**
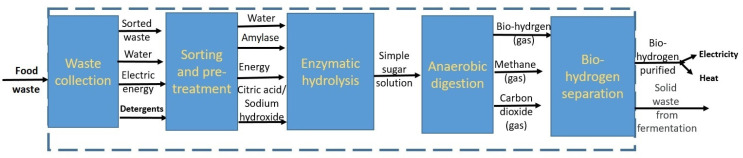
Block diagram illustrating the detailed process of biohydrogen production from food waste, including collection, preprocessing, enzymatic hydrolysis, fermentation, and hydrogen recovery, showcasing a sustainable approach to energy generation and waste reduction.

Biohydrogen production from food waste offers a sustainable approach to energy generation by utilizing organic waste as a feedstock. Through processes like dark fermentation, photofermentation, or hybrid methods, biodegradable materials are converted into hydrogen gas, reducing waste disposal issues while contributing to renewable energy development. A block diagram illustrating the detailed steps involved in the process of producing biohydrogen from food waste is presented in Figure 5. This diagram outlines the key stages, including the collection and preprocessing of food waste, enzymatic hydrolysis, fermentation, and final biohydrogen recovery, providing a clear overview of the conversion pathway.

Another valuable application is the production of bioactive peptides through enzymatic hydrolysis of protein-rich food waste, including dairy byproducts and meat processing residues. These peptides exhibit various biofunctional properties, such as antioxidant, antimicrobial, and antihypertensive activities, making them highly desirable in the health and nutrition industries [30,32].

Similarly, organic acids, such as lactic acid, citric acid, and succinic acid are produced from food waste through microbial fermentation processes. These acids have versatile applications in various industries, including food preservation, pharmaceuticals, biodegradable plastics, and cosmetics. For example, lactic acid is a precursor of polylactic acid (PLA), a biodegradable polymer used in sustainable packaging solutions.

Beyond these specific examples, food waste can also be transformed into secondary products, such as biofertilizers and animal feed, further extending its utility and contributing to a circular economy. The conversion of food waste into biofertilizers enriches the soil with essential nutrients, promotes sustainable agricultural practices, and reduces the need for chemical fertilizers. Similarly, processing food waste into animal feed not only addresses waste disposal challenges but also supports livestock production by providing an alternative and cost-effective source of nutrition. These transformations highlight the versatility of food waste as a resource, enabling the recovery of value while minimizing environmental impact and fostering a more sustainable and efficient resource management system.

## 4. Evaluating the Sustainability of Food Waste Management Systems

To address these challenges, sustainable food waste management systems are essential. Traditional disposal methods, such as landfilling and incineration, are no longer viable solutions due to their detrimental environmental and health impacts. Instead, modern systems focus on waste prevention, resource recovery, and the creation of circular economies. In a circular economy, food waste is viewed as a valuable resource that can be transformed into useful products, such as compost, bioenergy, or raw materials for industrial applications. This approach not only minimizes waste but also fosters economic opportunities and environmental benefits [31,40].

Evaluating the sustainability of food waste management systems is a critical step in ensuring their effectiveness and alignment with global sustainability goals. Such evaluations provide insights into the environmental, economic, and social performance of various strategies, enabling stakeholders to identify best practices, improve inefficiencies, and prioritize interventions. They also support compliance with international frameworks, such as the United Nations Sustainable Development Goals (SDGs), particularly Target 12.3, which aims to halve food waste per capita by 2030 [23,25].

The complexity of food waste management systems requires the use of advanced methodologies to comprehensively assess their sustainability (Table 4). These methodologies include life cycle assessment (LCA), which evaluates environmental impacts across the entire lifecycle of waste; cost-benefit analysis (CBA), which assesses economic feasibility; and material flow analysis (MFA), which tracks the flow of resources through the system. Additionally, sustainability assessment frameworks integrate environmental, social, and economic dimensions, offering a holistic evaluation approach [11,15].

## 5. Research Goals and Framework

This research focuses on analyzing and developing sustainable approaches for managing food waste, emphasizing the conversion of waste into valuable products, such as bioethanol, bioactive peptides, and organic acids. By employing a cost-benefit analysis (CBA), this study aims to evaluate the economic feasibility and environmental impact of these processes, offering a comprehensive framework to assess operational costs alongside economic and environmental advantages.

Food waste is a critical global issue with profound environmental consequences. Its improper management contributes to the generation of greenhouse gases, such as methane, which exacerbates climate change. Additionally, food waste leads to the unsustainable consumption of natural resources, including water, energy, and agricultural land, which are already under considerable strain globally [56,57,58]. Addressing this issue within the framework of a circular economy has emerged as a crucial strategy. By transforming food waste into valuable products, this approach not only minimizes waste but also ensures the reuse of resources, aligning with sustainable development objectives.

One of the key products derived from food waste is bioethanol, a renewable energy source that plays a vital role in reducing dependency on fossil fuels. Its production involves fermenting carbohydrate-rich food waste, such as starchy residues and fruit peels, using microbial processes. The use of bioethanol as an energy source significantly contributes to lowering CO_2_ emissions, addressing both energy security and environmental sustainability challenges [58,59]. Another promising product is bioactive peptides, which are obtained through the enzymatic hydrolysis of protein-rich food waste, including dairy byproducts and meat processing residues. These peptides exhibit various biofunctional properties, such as antioxidant and antihypertensive activities, making them highly valuable in the health and nutrition industries. They are increasingly utilized in the development of dietary supplements and functional foods, offering substantial health benefits while enhancing the value of food waste [60,61].

Additionally, organic acids are another valuable product obtained from food waste through microbial fermentation. These include lactic acid, citric acid, and succinic acid, which have applications in multiple industries. For instance, lactic acid serves as a precursor for the production of biodegradable plastics like polylactic acid (PLA), contributing to the development of sustainable packaging solutions. Similarly, organic acids play a pivotal role in food preservation and pharmaceutical formulations, demonstrating the versatility of food waste-derived products [62,63].

To evaluate the economic and ecological impacts of these processes, this study employs a cost-benefit analysis framework. This approach enables the identification of the most cost-effective and resource-efficient solutions, maximizing both economic returns and environmental benefits. By integrating these methodologies, this study provides actionable insights into the practical implementation of these technologies, ensuring their alignment with broader sustainability objectives. The successful deployment of these solutions can substantially reduce food waste while fostering the transition to a circular economy in which waste materials are repurposed as resources for the production of new goods [64,65].

This research ultimately aims to contribute to the establishment of a sustainable food waste management framework that not only addresses economic demands but also safeguards environmental resources and promotes public health. By advancing innovative waste-to-product conversion strategies, this study supports the achievement of global sustainability goals, demonstrating the potential of circular economy principles to mitigate pressing environmental challenges and drive sustainable development [66,67].

## 6. Cost-Benefit Analysis: Historical Development, Methodology, and Applications in Food Waste Valorization

Cost-benefit analysis (CBA) is a quantitative tool used in the economic, social, and financial assessment of the environmental study to identify the most suitable alternative from a series of alternatives [67]. Jules Dupuit, a world-famous French engineer, is considered the founder of CBA. In 1844, he proposed the idea of evaluating public projects (such as bridges or roads) by analyzing the benefits they bring to their users in comparison with their costs [68]. During 1960–1980, CBA was adopted and developed by international organizations, such as the World Bank, the UNO, and national governments, for the evaluation of their large projects, including economic development and infrastructure projects [69]. In addition, during this period, new elements were introduced in the CBA methodology, such as discounting, to assess the long-term value of projects [70].

In addition to the economic aspects, the analysis also includes impacts on the environment or natural resources [71]. CBA also functions as a decision-making tool by comparing the costs and benefits associated with the research study to assess the overall impacts of the study [72].

Recent literature provides some relevant examples of studies that have used cost-benefit analysis (CBA) to evaluate enzymatic hydrolysis in sustainable food waste management. Some studies have analyzed the economic feasibility and environmental benefits of converting food waste into products, such as bioethanol, through enzymatic hydrolysis in their study. Others have focused their research on improving ultrasonic enzymatic hydrolysis to produce glucose from food waste. In addition, the use of enzymatic hydrolysis in combination with anaerobic digestion for biogas production was evaluated. The study of biohydrogen production from food waste using separate hydrolysis and fermentation highlighted the sustainability of this process for the efficient conversion of waste carbohydrates to biohydrogen. Some research has evaluated the efficiency of enzymatic hydrolysis combined with ultrasound to obtain glucose, a bioethanol precursor, using food waste. The economic analysis included operational costs and environmental benefits. Other studies have focused on the use of enzymatic hydrolysis in tandem with anaerobic digestion for biogas production from food waste, applying CBA to assess economic feasibility [73,74,75,76,77,78].

In the context of the circular economy, applying CBA for the production of bio-based products and advanced biofuels is essential for optimizing the use of resources and minimizing environmental impacts. The circular economy emphasizes the importance of recycling materials and energy, promoting a sustainable development model that minimizes waste, and encourages its reuse [79,80].

### 6.1. Steps for the Assessment of Food Waste Reduction and Recovery Scenarios, Estimation of Economic and Environmental Efficiency Through Cost-Benefit Analysis (CBA)

The primary objective of employing the cost-benefit analysis (CBA) methodology in this study was to evaluate the economic, social, and environmental impacts of the enzymatic hydrolysis process. This process facilitates the breakdown of complex organic materials, such as carbohydrates, proteins, and lipids, into simpler compounds for their conversion into valuable biofuels and bioproducts. By implementing CBA, we aimed to assess the cost-effectiveness of these practices, considering both initial and operational expenditures alongside the long-term benefits of economic viability, environmental sustainability, and social advantages.

**Figure 6:**
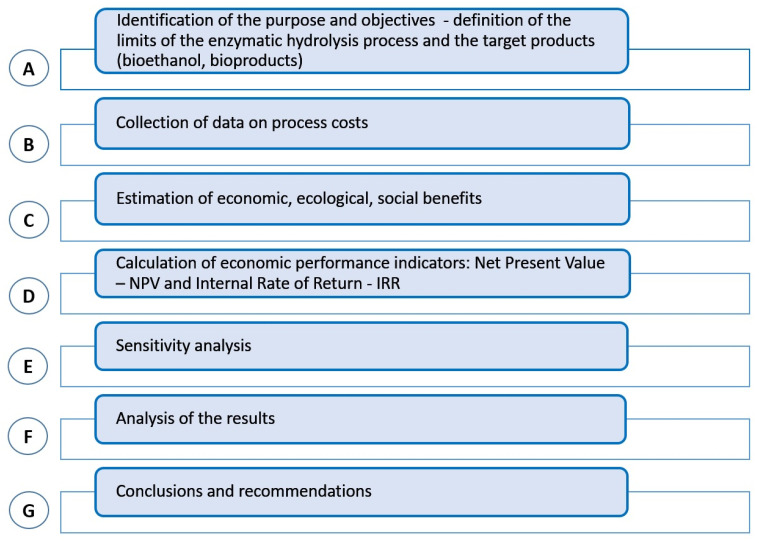
Steps followed to apply cost-benefit analysis (CBA), outlining the sequential process used to evaluate the economic feasibility of alternatives, including the identification of costs and benefits, data collection, ratio calculations, and decision-making criteria for scenarios A1–A3.

To conduct CBA for enzymatic hydrolysis of food waste, we adhered to a structured approach that encompassed several essential steps (Figure 6). These steps involve quantifying the costs associated with enzyme procurement, energy consumption, and process optimization, as well as evaluating the generated benefits, including reduced waste, lower greenhouse gas emissions, and the production of renewable biofuels and high-value bioproducts [56]. This comprehensive analysis offers insights into the scalability and practicality of this approach for addressing food waste challenges and fostering sustainable resource utilization.

Consequently, the cost-benefit analysis (CBA) methodology has been employed to evaluate the economic, social, and environmental impacts of methods aimed at food waste recovery, resource conservation, and the generation of commercial products. This approach enables a systematic assessment of the financial investments required and the broader benefits achieved, such as reduced environmental degradation, efficient resource utilization, and the creation of marketable products. By quantifying these factors, CBA provides valuable insights into the feasibility and sustainability of strategies to address food waste and enhance resource recovery.

### 6.2. Defining Application Limits and Scenarios

In developing limits and scenarios for the use of enzymes to degrade carbohydrates, proteins, and lipids from food waste into simpler compounds with the potential for valorization across various industries, it is important to evaluate the available alternatives, considering their respective advantages and limitations. This process involves a comprehensive analysis of technological feasibility, economic viability, and environmental impact to ensure that the most effective approach is selected for implementation. Each scenario requires careful assessment to optimize the enzymatic hydrolysis process and balance efficiency, cost-effectiveness, and environmental sustainability.

✓ **Scenario A1:** Bioethanol Production via Enzymatic Hydrolysis of Food Waste

The production of bioethanol through enzymatic hydrolysis of food waste follows a structured sequence of stages, each essential for converting organic material into a renewable energy source.

- Raw material preparation

Food waste (e.g., vegetable and fruit peelings, off-cuts, unusable vegetable and fruit scraps, old bread, cakes, unusable milk or cheese, unused potatoes, corn, or rice) is collected, sorted, and pre-treated to remove impurities and reduce particle size. This preprocessing facilitates enzyme access to the substrate and enhances hydrolysis efficiency [81].

b. Enzymatic hydrolysis stage

The enzymatic hydrolysis of food waste for bioethanol production is divided into several sub-stages [82,83,84,85,86]:

- Substrate preparation: Food waste is shredded, dried, or heat-pretreated to increase substrate accessibility for enzymes.
- Enzyme addition: Specific enzymes, such as cellulases, amylases, and proteases, are introduced to degrade complex carbohydrates, proteins, and lipids into simpler compounds.
- Hydrolysis process: Enzymes act on the chemical bonds within large molecules (e.g., starch or cellulose), breaking them into simple sugars like glucose and fructose.
- Optimization of reaction conditions: Proper temperature, pH, and reaction time are controlled to maximize hydrolysis efficiency.
- Process monitoring: The hydrolysis progress is regularly observed to ensure process consistency.

c. Fermentation stage

After enzymatic hydrolysis, simple sugars obtained from food waste are converted into bioethanol through fermentation by microorganisms (e.g., yeasts). This stage consists of the following sub-steps [87,88,89,90,91]:

- Inoculation with microorganisms: Yeast (e.g., *Saccharomyces cerevisiae*) is added to the substrate containing sugars from hydrolysis.
- Fermentation process: Microorganisms metabolize sugars, producing bioethanol and carbon dioxide. The optimum temperature is maintained at 30–35 °C.
- Process monitoring: The bioethanol concentration, fermentation time, and pH are closely monitored to ensure process efficiency.
- Completion of fermentation: This process continues until all available sugars are consumed, yielding a high concentration of ethanol.
- Separation and purification: The fermentation mixture is separated from the solid residues via filtration or centrifugation, and the bioethanol is further purified through distillation.

d. Distillation and purification stage

The bioethanol resulting from fermentation undergoes the following steps for purification [92,93,94,95,96]:

- Pre-distillation: The fermentation mixture containing bioethanol, water, and other substances is heated in a distiller. Ethanol, which has a lower boiling point than water, evaporates first.
- Condensation: The ethanol vapors are condensed in a cooling system and collected in a separate container.
- Further distillation: To increase the ethanol concentration, the distillation process may be repeated or extended in a distillation column, separating ethanol vapor from residual water.
- Dehydration: To remove any remaining water, additional dehydration methods, such as zeolite adsorption or azeotropic distillation, are applied.
- Testing and standardization: The final ethanol product is tested to ensure compliance with purity standards. The ethanol concentration is monitored before use or marketing.

✓ **Scenario A2:** Bioactive Peptide Production via Enzymatic Hydrolysis of Food Waste

The process of obtaining bioactive peptides from enzymatic hydrolysis of food waste involves the breakdown of proteins into smaller components, known as peptides, through the use of specific enzymes. This approach transforms food waste into a valuable resource for the food and health industries, as the peptides obtained can be utilized in a variety of applications, including the formulation of dietary supplements and functional foods [97].

The steps of the enzymatic hydrolysis process are as follows:

1. Raw material selection and preparation

Food waste, including vegetables, fruits, cereals, or meat products, is collected and prepared as raw material. The collected waste is cleaned, and depending on its characteristics, it may be shredded to improve enzyme accessibility to proteins. This preparation stage ensures optimal substrate quality for enzymatic hydrolysis [98].

2. Enzyme activity

The proteins in food waste are subjected to proteolytic enzymes, such as proteases, which catalyze the cleavage of peptide bonds between amino acids. The type and concentration of the enzyme are carefully selected and adjusted based on the specific nature of the substrate and the desired process outcomes, such as obtaining longer or shorter peptide chains. This stage is crucial for achieving the targeted bioactive properties of the peptides [99].

3. Peptide purification

Following the enzymatic reaction, the enzyme solution undergoes filtration and treatment to remove insoluble particles. Bioactive peptides are then separated and purified using methods such as ultrafiltration, chromatography, or precipitation. These peptides are subsequently analyzed to determine their quality, concentration, and suitability for specific applications, ensuring the production of high-value bioactive compounds [100].

✓ **Scenario A3:** Organic Acid Production via Enzymatic Hydrolysis of Food Waste

The production of organic acids through enzymatic hydrolysis of food waste involves the breakdown of complex organic components, such as carbohydrates and proteins, into simpler compounds that are further converted into organic acids. These organic acids are valuable for various industries, including food, pharmaceuticals, and cosmetics, due to their preservative properties and applications in the synthesis of new products [101].

The steps involved in obtaining organic acids by enzymatic hydrolysis are as follows:

1. Preparation of raw materials

Food waste, such as vegetable, fruit, cereal, and bakery product scraps, is collected and cleaned for use in the hydrolysis process. If the waste is large or dense, it can be shredded or heat-treated to enhance enzyme accessibility. This preprocessing ensures optimal conditions for efficient enzymatic action [102].

2. Enzyme selection and application

Specific enzymes, such as amylases, proteases, and lipases, are used in the hydrolysis process to break down carbohydrates, proteins, and lipids, respectively. The choice of enzyme depends on the type of food waste and the desired organic acids, such as lactic acid, acetic acid, or butyric acid. These enzymes catalyze the decomposition of complex molecules into target organic acids, enabling a highly controlled and efficient conversion process [103].

3. Analysis and use of organic acids

The organic acids obtained are analyzed to determine their purity and concentration. Based on their quality, these acids are then used in various applications, such as food preservation in the food industry, drug formulation and supplements in the pharmaceutical industry, and other industrial uses. This stage ensures the production of high-quality organic acids tailored to meet specific industry needs [104].

### 6.3. Development of the Second Step B: Determining and Assessing Costs and Benefits

The step of determining and evaluating costs is a critical component of scenario analysis, particularly for processes such as the production of bioethanol, peptides, and fatty acids. This step involves a systematic approach to identifying, quantifying, and analyzing associated costs and benefits. The key elements of this step include the following:

- Cost identification
- Cost actualization
- Benefits identification
- Benefits actualization
- Calculation of cost-benefit and benefit-cost ratios

- Identification of costs associated with the three scenarios (A1–A3)

1. Implementation costs

These costs include the procurement and installation of the equipment required for the production process.

2. Maintenance process costs

These include expenses for staff salaries, energy consumption for equipment operation, enzymes used in enzymatic hydrolysis, maintenance costs, and management of secondary waste generated during the process.

3. Post-implementation costs

These refer to ongoing expenditures related to process monitoring, efficiency evaluation, further research and development activities, and compliance with reporting and legislative requirements.

- Actualization of costs

The actualization of costs was conducted over a 10-year period using an actualization rate of 5%. The actualization factor was calculated using Equation (1) [105,106]:

*fa* = 1/(1 + *r*)*^t^* (1)

where: *fa*—is the actualization factor, *r*—is the discount rate, and *t*—is the recovery year of the investment.

This structured approach ensures a comprehensive assessment of the financial feasibility and sustainability of the proposed scenarios, aiding in informed decision-making for project implementation.

The following values result from the actualization factor [107,108,109]:
*f_a_*^1^ = 1/(1 + 0.05)^1^ = 0.952 *f_a_*^2^ = 1/(1 + 0.05)^2^ = 0.907 *f_a_*^3^ = 1/(1 + 0.05)^3^ = 0.863 *f_a_*^4^ = 1/(1 + 0.05)^4^ = 0.822 *f_a_*^5^ = 1/(1 + 0.05)^5^ = 0.783*f_a_*^6^ = 1/(1 + 0.05)^6^ = 0.746 *f_a_*^7^ = 1/(1 + 0.05)^7^ = 0.710 *f_a_*^8^ = 1/(1 + 0.05)^8^ = 0.676 *f_a_*^9^ = 1/(1 + 0.05)^9^ = 0.644 *f_a_*^10^ = 1/(1 + 0.05)^10^ = 0.613

The direct cost values, which represent the expenses that are directly associated with implementing the selected scenarios, are presented in Table 5, Table 6 and Table 7. These costs are tangible, easily identifiable, and directly linked to the implementation of scenarios ([107,108,109], https://ec.europa.eu/eurostat/statistics-explained/index.php?title=Construction_producer_price_and_construction_cost_indices_overview (accessed on 1 December 2024); https://constructioncosts.eu/ (accessed on 1 December 2024); https://www.statista.com/topics/10928/construction-costs-in-europe 1st of December, 2024/ (accessed on 1 December 2024); https://tradingeconomics.com/european-union/construction-cost-idx-eurostat-data.html (accessed on 1 December 2024); https://www.ecb.europa.eu/stats/macroeconomic_and_sectoral/hicp/html/index.en.html (accessed on 1 December 2024)).

Table 8, Table 9 and Table 10 include costs actualization data, which refers to the process of adjusting costs to reflect their present value, accounting for the time value of money. This ensures that costs incurred or projected at different points in time are accurately compared in terms of their equivalent value today. This is particularly important in scenarios that span several years, as the purchasing power of money changes over time due to inflation, interest rates, and other economic factors.

**Table 5:** Direct costs associated with scenario A1.

Cost Type		Cost Value for A1 (EUR)	Total
			(EUR)
Initial costs for implementation	Pre-treatment equipment	20,000	745,000
	Enzymatic hydrolysis equipment	300,000	
	Fermentation and distillation reactors	400,000	
	Purification and separation plants	10,000	
	Enzymes for hydrolysis	5000	
	Transportation and logistics costs	4000	
	Legal and license costs	6000	
Operation and maintenance costs	Employment costs (salaries)	40,000	105,000
	Energy costs (electricity, heat)	30,000	
	Equipment maintenance costs	10,000	
	Operating enzyme costs	6000	
	Operating costs of distillation and fermentation equipment	16,000	
	Secondary waste management costs	3000	
Post-implementation costs	Monitoring and continuous evaluation costs	5000	50,000
	Reporting and compliance costs	10,000	
	Product development: creating new applications or products derived from starch	20,000	
	Capacity extension: expanding infrastructure to increase production volume	15,000	
Total			900,000

**Table 6:** Direct costs associated with Scenario A2.

Cost Type		Cost Value for A2 (EUR)	Total
			(EUR)
Initial costs for implementation	Pre-treatment equipment	16,000	92,000
	Enzymatic hydrolysis equipment	24,000	
	Fermentation and distillation reactors	30,000	
	Purification and separation of plants	10,000	
	Enzymes for hydrolysis	6000	
	Transportation and logistics costs	2000	
	Legal and license costs	4000	
Operation and maintenance costs	Employment costs (salaries)	20,000	68,000
	Energy costs (electricity, heat)	8000	
	Equipment maintenance costs	4000	
	Operating enzyme costs	12,000	
	Operating costs of distillation and fermentation equipment	24,000	
	Secondary waste management costs	1600	
Post-implementation costs	Monitoring and continuous evaluation costs	5000	14,600
	Reporting and compliance costs	3000	
	Product development: creating new applications or products derived from starch	1000	
	Capacity extension: expanding infrastructure to increase production volume	5600	
Total			174,600

**Table 7:** Direct costs associated with Scenario A3.

Cost Type		Cost Value for A3 (EUR)	Total
			(EUR)
Initial costs for implementation	Pre-treatment equipment	14,000	108,000
	Enzymatic hydrolysis equipment	30,000	
	Fermentation and distillation reactors	36,000	
	Purification and separation of plants	12,000	
	Enzymes for hydrolysis	8000	
	Transportation and logistics costs	3000	
	Legal and license costs	5000	
Operation and maintenance costs	Employment costs (salaries)	36,000	93,000
	Energy costs (electricity, heat)	24,000	
	Equipment maintenance costs	10,000	
	Operating enzyme costs	6000	
	Operating costs of distillation and fermentation equipment	14,000	
	Secondary waste management costs	3000	
Post-implementation costs	Monitoring and continuous evaluation costs	1000	10,000
	Reporting and compliance costs	2000	
	Product development: creating new applications or products derived from starch	4000	
	Capacity extension: expanding infrastructure to increase production volume	3000	
Total			211,000

In these tables, the symbols have the following significance:

*C_it_*—the initial costs, which represent the costs incurred at the beginning of the project (e.g., equipment purchase, installation, or setup expenses),

*C_expt_*—operational or ongoing costs, which refer to the expected or recurring costs associated with maintaining and operating the project over time (e.g., energy consumption, labor, raw materials).

*C_t_* = *C_it_* + *C_expt_*—total costs, which represent the total cost in a specific time period *t*, combining both initial costs (*C_it_*) and expected operational costs (*C_exp_*).

*f_a_*—actualization factor, calculated with Equation (1), used to adjust costs to their present value

*C_at_* = *C_t_* × *f_a_*—actualized cost, representing the total cost (*C_t_*) adjusted for the time value of money using the actualization factor (*f_a_*). This gives the present value of the cost at time *t*.

### 6.4. Estimation of Economic, Ecological, Social Benefits

The third step of the cost-benefit analysis involves estimating the economic, ecological, and social benefits generated by the proposed scenarios. This step is crucial for a comprehensive evaluation, as it not only quantifies financial gains, but also considers contributions to environmental sustainability and societal well-being. By integrating these dimensions, the analysis provides a thorough understanding of the scenarios’ overall impacts and their alignment with broader project objectives.

**Table 8:** Costs actualization associated with alternative (A1).

Year (t)	C_it_	C_expt_	C_t_ = C_it_ + C_expt_	f_a_	C_at_ = C_t_ x f_a_
1	115,000	0	115,000	0.95	109,250
2	0	105,000	105,000	0.91	95,550
3	0	105,000	105,000	0.86	90,300
4	0	105,000	105,000	0.82	86,100
5	0	105,000	105,000	0.78	81,900
6	0	105,000	105,000	0.75	78,750
7	0	105,000	105,000	0.71	74,550
8	0	105,000	105,000	0.68	71,400
9	0	105,000	105,000	0.64	67,200
10	10,500	0	10,500	0.61	6405
Total					761,405

**Table 9:** Costs actualization associated with alternative (A2).

Year (t)	C_it_	C_expt_	C_t_ = C_it_ + C_expt_	f_a_	C_at_ = C_t_ x f_a_
1	92,000	0	92,000	0.95	87,400
2	0	68,000	68,000	0.91	61,880
3	0	68,000	68,000	0.86	58,480
4	0	68,000	68,000	0.82	55,760
5	0	68,000	68,000	0.78	53,040
6	0	68,000	68,000	0.75	51,000
7	0	68,000	68,000	0.71	48,280
8	0	68,000	68,000	0.68	46,240
9	0	68,000	68,000	0.64	43,520
10	14,600	0	14,600	0.61	8906
Total					514,506

**Table 10:** Costs actualization associated with alternative (A3).

Year (t)	C_it_	C_expt_	C_t_ = C_it_ + C_expt_	f_a_	C_at_ = C_t_ x f_a_
1	108,000	0	108,000	0.95	102,600
2	0	93,000	93,000	0.91	84,630
3	0	93,000	93,000	0.86	79,980
4	0	93,000	93,000	0.82	76,260
5	0	93,000	93,000	0.78	72,540
6	0	93,000	93,000	0.75	69,750
7	0	93,000	93,000	0.71	66,030
8	0	93,000	93,000	0.68	63,240
9	0	93,000	93,000	0.64	59,520
10	10,000	0	10,000	0.61	6100
Total					680,650

- Benefits identification

The benefits of scenarios A1–A3 have been categorized into three key domains: economic, environmental, and social. These domains encompass not only physical and ecological indicators, such as resource efficiency and environmental impact mitigation, but also socioeconomic variables, which can be quantitatively assessed to provide a comprehensive evaluation. The direct economic benefits have been calculated to represent 5% of the total costs, offering a tangible measure of immediate financial advantages. The net present value (NPV) of these benefits has been determined by employing a discount rate equal to the inflation rate over a discount period of 10 years, ensuring a realistic and time-adjusted valuation of the benefits. These findings are summarized in Table 11.

Additionally, Table 12 provides a broader estimate of the social and environmental benefits associated with each process type for producing bioethanol, peptides, and organic acids. These benefits include improvements in waste management, reduced greenhouse gas emissions, and potential socioeconomic contributions, such as job creation and energy security, highlighting the multifaceted advantages of the proposed processes.

- Benefits actualization

Actualized benefits represent the present value of benefits anticipated over a 10-year period, adjusted to account for the time value of money. This calculation involves discounting future benefits to their present value using an appropriate discount rate, ensuring that the benefits realized at different points in time are evaluated on a consistent economic basis. By considering the time-dependent value of benefits, this approach provides a more accurate and comparable assessment of long-term impacts.

Table 13 illustrates the actualized benefits calculated for the 10-year period associated with the three scenarios, A1, A2, and A3. This analysis highlights the economic viability of each scenario by providing a clear comparison of their respective long-term benefits in monetary terms, offering valuable insights for decision-making and the prioritization of sustainable practices.

**Table 11:** Identification of the direct social and environmental benefits associated with scenarios A1–A3.

Type of Benefit	Bioethanol	Peptides	Organic Acids
Economic	Direct benefits (5% of total costs)	Direct benefits (5% of total costs)	Direct benefits (5% of total costs)
Social	- Creation of jobs in the green sector	- Increased economic opportunities in the food and pharmaceutical industries	- Development of sustainable industries and markets for bio-based products
	- Increased energy security through renewable sources	- Improvement of the quality of food and nutraceutical products	- Greater access to eco-friendly chemical products for various industries
	- Reduction of dependence on fossil fuels	- Support for research and innovation in biotechnology	- Promotion of local and regional development through the use of available resources
Environmental	- Reduction of CO_2_ emissions and greenhouse gases	- Minimization of environmental impact by using organic waste	- Pollution reduction by recycling organic waste
	- Efficient management of organic waste and reduction of landfill waste	- Use of renewable resources and reduction of natural raw material consumption	- Conservation of natural resources and reduction of chemical pollution
	- Contribution to reduced air pollution and improved air quality	- Use of eco-friendly processes with low energy consumption	- Promotion of recycling and reuse of resources for the production of eco-friendly chemicals

**Table 12:** The estimated amounts for the economic, social, and environmental benefits for scenarios A1–A3.

Scenario	Economic Benefits (EUR/Year)	Social Benefits (EUR/Year)	Environmental Benefits (EUR/Year)	Total (EUR/Year)
A1	11,525	430,000	725,000	1,166,525
A2	8730	315,000	564,000	887,730
A3	10,550	280,000	425,000	715,550

**Table 13:** Actualization of benefits resulting from the A1–A3 implementation.

Year	Annual Benefits (Ba) (EUR)	Actualization Factor (f_a_)	Actualized Benefits (B_at_) A1 (EUR)	Actualized Benefits (B_at_) A2 (EUR)	Actualized Benefits (B_at_) A3 (EUR)
1	0	0.952	0	0	0
2	1,166,525	0.907	1,058,038.18	805,171.11	649,003.85
3	1,166,525	0.863	1,006,711.08	766,110.99	617,519.65
4	1,166,525	0.822	958,883.55	729,714.06	588,182.1
5	1,166,525	0.783	913,389.075	695,092.59	560,275.65
6	1,166,525	0.746	870,227.65	662,246.58	533,800.3
7	1,166,525	0.71	828,232.75	630,288.3	508,040.5
8	1,166,525	0.676	788,570.9	600,105.48	483,711.8
9	1,166,525	0.644	751,242.1	571,698.12	46,0814.2
10	0	0.613	0	0	0
Total			7,175,295.28	5,460,427.23	4,401,348.05

**Figure 7:**
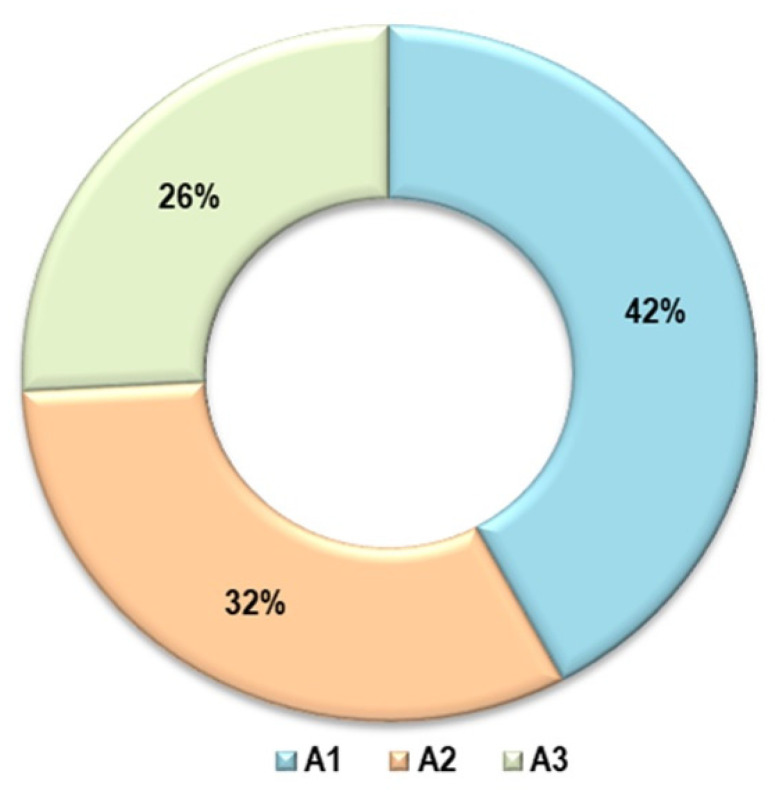
Percentage of actualized benefits for alternatives A1–A3, illustrating the proportion of realized benefits relative to potential outcomes, reflecting their varying levels of efficiency and effectiveness in benefit realization.

Figure 7 provides a clear visual comparison of the actualized benefit rates for the three food waste management alternatives, A1, A2, and A3, over a 10-year period. The percentages of 42% for A1, 32% for A2, and 26% for A3 illustrate the distribution of the present value of benefits across these scenarios. These percentages are derived by discounting future benefits to their present value, ensuring that all benefits occurring over the time horizon are assessed on an equivalent economic basis, as described in the text.

The data in Figure 7, when viewed alongside Table 13, underscores that alternative A1 delivers the highest actualized benefits at 42%, followed by A2 and A3 at 32% and 26%, respectively. This suggests that A1 is the most economically advantageous option among the three, offering the greatest return when future benefits are adjusted for the time value of money. By highlighting these differences, Figure 7 helps to emphasize the relative effectiveness of each alternative in achieving long-term benefits, which can aid decision makers in selecting the most viable food waste management strategy.

- Calculation and analysis of cost-benefit (C/B) and benefit-cost (B/C) ratios

Cost-benefit and benefit-cost ratios are essential analytical tools for evaluating the economic viability of proposed scenarios, providing a systematic framework for comparing costs and benefits. These metrics quantify the trade-offs between the resources invested and the outcomes achieved, offering a comprehensive perspective on the financial and economic efficiency of each scenario.

The cost-benefit ratio (C/B Ratio) focuses on the proportion of benefits to costs, illustrating the overall value created relative to the resources expended. This ratio helps determine whether the investment in a scenario justifies the expected benefits. Meanwhile, the benefit-cost ratio (B/C Ratio) measures the amount of benefit generated for every unit of cost, shedding light on the efficiency of the allocation of resources within each scenario. By quantifying this relationship, these tools enable a detailed assessment of potential economic returns in different scenarios.

These ratios not only aid in identifying scenarios that deliver significant financial advantages but also help in prioritizing options that align with strategic goals, such as enhancing sustainability, reducing waste, or maximizing social and environmental benefits. The application of these metrics is decisive in guiding decision makers toward scenarios that optimize both economic returns and broader societal impacts, providing a robust basis for long-term planning and resource allocation.

The benefit-to-cost (B/C) ratio is the sum of the total benefits obtained from the scenario implementation divided by the total costs associated with its implementation. It is calculated using Equation (2).

B/C Ratio = (Total Benefits)/(Total Costs)(2)

A ratio greater than 1 signifies that the benefits exceed the costs, making the scenario financially attractive. A ratio less than 1 suggests that the costs are higher than the benefits, potentially making the scenario unviable. A ratio equal to 1 means that the scenario is economically neutral (benefits equal costs).

The cost-benefit Ratio (C/B) is the sum of the total costs associated with the scenario divided by the sum of the total benefits (Equation (3)).

C/B Ratio = (Total Costs)/(Total Benefits)(3)

A ratio greater than 1 signifies that the benefits exceed the costs, making the scenario financially attractive. A ratio less than 1 suggests that the costs are higher than the benefits, potentially making the project unviable. A ratio equal to 1 means that the scenario is economically neutral (costs equal to benefits).

- Cost-benefit and benefit-cost ratios

**Figure 8:**
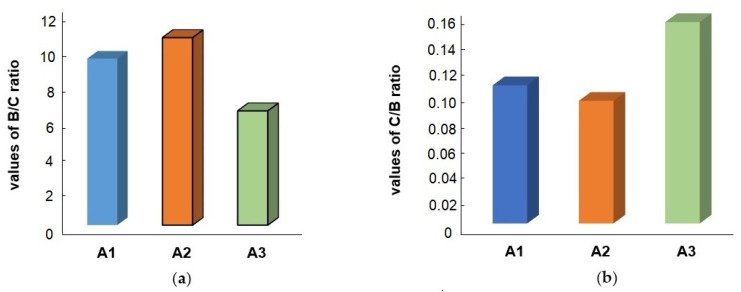
Results of (**a**) benefit-cost (B/C) ratio analysis, highlighting the relative advantages gained per unit cost for scenarios A1–A3, and (**b**) cost-benefit (C/B) ratio analysis, emphasizing the cost incurred per unit benefit for scenarios A1–A3, providing a comprehensive assessment of economic efficiency and viability across the alternatives.

The cost-benefit and benefit-cost ratios are essential indicators for evaluating the economic viability of different alternatives. Figure 8 illustrates the results of the analysis of the cost-benefit (C/B) and benefit-cost (B/C) ratios, providing a comprehensive comparison of the economic performance of scenarios A1–A3. These ratios evaluate the cost efficiency and benefits achieved per unit cost, providing valuable insights into the relative economic viability of each alternative.

✓ Benefit-Cost (B/C) Ratio

The benefit-cost (B/C) ratio serves as a critical metric for assessing the economic efficiency of different scenarios by quantifying the benefits obtained per unit of cost incurred. A higher B/C ratio reflects a more favorable outcome, as it indicates that greater benefits are derived from the same level of expenditure, which is essential in guiding decision makers toward more economically sound investments.

Among the evaluated scenarios, A2 stands out with a B/C ratio of 10.613, which is the highest among the three. This indicates that scenario A2 yields significantly greater benefits than its costs, positioning it as the most economically viable option. The high ratio demonstrates that A2 not only maximizes returns on investment but also represents the most efficient allocation of resources for achieving the intended benefits. Consequently, A2 offers the best potential for long-term economic sustainability, making it a highly attractive choice for implementation.

In contrast, A3 records the lowest B/C ratio at 6.466, indicating that it produces comparatively fewer benefits per unit of cost. This lower ratio suggests that A3 may be less effective in delivering economic returns and may require further optimization to improve its performance. Factors such as operational inefficiencies, higher costs, or limited benefits could contribute to its lower economic appeal. To enhance A3’s viability, efforts could focus on cost reduction or increasing the associated benefits through strategic adjustments or process improvements.

A1, with a B/C ratio between those of A2 and A3, offers a balanced performance, making it a moderate alternative. While it does not outperform A2 in terms of benefit generation, it still provides a reasonable return on investment and is more advantageous than A3. This positions A1 as a stable and reliable option, particularly in cases where extreme optimization (as seen in A2) is not feasible or desirable. The intermediate performance of A1 ensures that it remains a viable choice, offering a compromise between high benefits and moderate costs.

The analysis of the B/C ratios highlights clear distinctions in the economic performance of the three scenarios. A2 emerges as the most beneficial and cost-effective alternative, A1 presents a balanced option with moderate returns, and A3, while less economically favorable, provides an opportunity for improvement through targeted interventions. These insights are valuable for decision makers aiming to prioritize investments that yield the highest economic benefits while ensuring cost efficiency.

✓ Cost-Benefit (C/B) Ratio

The cost-benefit (C/B) ratio is a key indicator of economic performance, representing the cost incurred per unit of benefit generated. A lower C/B ratio is more desirable, as it indicates higher cost efficiency, meaning that the same level of benefits is achieved with reduced expenditure. This ratio is crucial for comparing the relative cost-effectiveness of different alternatives, helping to identify the most efficient use of resources.

Among the three scenarios evaluated, A2 exhibits the lowest C/B ratio of 0.094, making it the most cost-efficient option. This result implies that A2 requires the least amount of expenditure to deliver its intended benefits, thereby maximizing cost savings. The superior performance of A2 in terms of cost efficiency highlights its economic attractiveness and potential for sustainable implementation. The combination of low costs and high benefits, as evidenced by its C/B and B/C ratios, underscores A2’s suitability for prioritization in food waste valorization strategies.

By contrast, A3, with a C/B ratio of 0.155, represents the least cost-efficient alternative among the three. The relatively high C/B ratio suggests that A3 incurs the highest cost to generate an equivalent level of benefit, reducing its overall economic appeal. This raises concerns regarding the feasibility of A3 without further optimization. To improve its cost efficiency, targeted measures, such as process optimization, cost reduction strategies, or enhanced benefit realization, could be considered. The high C/B ratio indicates the need for additional analysis to identify potential inefficiencies or areas where improvements could lead to better economic performance.

A1 lies between A2 and A3, offering a moderate C/B ratio that reflects a balanced cost-efficiency level. While A1 does not achieve the outstanding cost-effectiveness of A2, it still performs better than A3, making it a viable middle-ground option. The reasonable balance between costs and benefits suggests that A1 could serve as a dependable alternative, especially in scenarios where maximizing cost savings is not the primary objective.

Overall, the comparative analysis of C/B ratios reinforces the superior economic performance of A2, which not only achieves the lowest cost per unit of benefit but also delivers the highest benefit per unit of cost, as indicated by its B/C ratio. This dual advantage positions A2 as the most economically viable option for food waste valorization. In contrast, A3 consistently ranks lowest in both measures, making it the least attractive alternative. The results suggest that while A3 may still have potential, significant improvements are necessary to enhance its cost efficiency and economic viability. A deeper review of A3’s processes and cost structure could help identify optimization opportunities.

The metrics clearly favor A2 as the optimal scenario, offering the greatest benefits at the lowest cost, while A1 serves as a balanced option with moderate efficiency. Meanwhile, A3, being the least favorable, requires further refinement to justify its implementation or improve its cost-effectiveness. These findings provide valuable guidance for decision makers in selecting and optimizing food waste valorization strategies.

### 6.5. Calculation of Economic Performance Indicators: Net Present Value—NPV and Internal Rate of Return—IRR and Sensitivity Analysis

#### 6.5.1. Net Present Value and Internal Rate of Return

In this step, two key economic performance indicators were calculated to evaluate the financial viability of the proposed scenarios: the internal rate of return (IRR) and net present value (NPV). These indicators provide a comprehensive assessment of a project’s profitability and resilience under varying conditions.

The IRR is defined as the discount rate at which the present value of a project’s future benefits equals the present value of its costs, resulting in a net present value of zero. It represents the rate of return a project generates based on its cash flow. The IRR was calculated using Equation (4) [107,108,109]:

(4) $$IRR=\left(\frac{VAN}{\mathrm{Initial}\;\mathrm{cost}}-1\right)\cdot 100\;(\%)$$

The IRR provides a useful benchmark for evaluating the financial viability of a project by comparing it with the cost of capital (the minimum required rate of return). Three possible situations may arise.

- IRR > cost of capital: when the IRR exceeds the cost of capital (minimum required rate of return), the scenario is deemed profitable and should be considered for implementation.
- IRR = cost of capital: when the IRR equals the cost of capital, the project is marginally profitable, sitting at the threshold of financial viability.
- IRR < cost of capital: when the IRR is lower than the cost of capital, the project is considered unprofitable and is unlikely to be a viable investment.

This indicator is particularly useful for comparing alternative scenarios to determine the scenario that provides the highest financial return relative to its cost.

The NPV is calculated as the difference between the present value of the project’s future cash inflows (benefits) and the present value of its cash outflows (costs). It represents the net economic value that a scenario contributes after accounting for the time value of money. The NPV was calculated using Equation (5) [107,108,109]:

(5) $$NPV=\sum_{t=1}^{n}\frac{C_{t}}{{\left(1+r\right)}^{t}}-C_{0}$$

where *C_t_* = net cash flow at time t; *r* = discount rate; *t* = point in time (year); *C*_0_ = the initial cost of the investment.

The net present value (NPV) helps determine the financial viability of a project by comparing the present value of its benefits with the present value of its costs. Three situations can be identified.

- NPV > 0: A positive NPV indicates profitability, meaning the present value of benefits exceeds the present value of costs. The project should be accepted.
- NPV = 0: A zero NPV signifies that the project is at the break-even point, where the benefits exactly match the costs. Such a project is marginally acceptable.
- NPV < 0: A negative NPV reflects unprofitability, indicating that costs outweigh benefits and that the project should likely be rejected.

The NPV provides a clear indication of a project’s value addition in monetary terms and is a key metric for investment decision-making.

#### 6.5.2. Sensitivity Analysis

Sensitivity analysis is a critical tool used to evaluate the robustness and resilience of a scenario’s financial and operational performance under varying conditions. By systematically altering key variables, such as costs, benefits, discount rates, or cash flow, sensitivity analysis assesses how these changes impact economic performance indicators like net present value (NPV) and internal rate of return (IRR). This approach identifies the variables to which a certain scenario is most sensitive, thereby providing valuable insights into the potential risks and uncertainties [107,108,109].

This analysis is particularly important for scenarios subject to fluctuating market conditions or unpredictable external factors. By examining both optimistic and pessimistic scenarios, sensitivity analysis helps determine the range of conditions under which a project remains viable. This ensures a more comprehensive evaluation of its feasibility, enabling stakeholders to make informed decisions and prepare mitigation strategies for potential risks. The objective of the sensitivity analysis was to evaluate how the variation in key parameters (amount of waste, implementation costs, technology efficiency, bioproduct prices, etc.) can influence the economic, environmental, and social results of the proposed solutions.

The sensitivity index is a quantitative measure used in sensitivity analysis to assess the degree to which variations in key input parameters impact the economic performance indicators of a scenario (NPV and IRR). It provides a systematic way to identify the critical factors influencing scenario viability by expressing the relative change in an output variable in response to a unit change in an input variable. This index is particularly valuable for evaluating the robustness of a scenario under different circumstances, such as optimistic and pessimistic conditions, which represent the best-case and worst-case conditions under which a scenario might operate. The optimistic scenario assumes favorable outcomes, such as reduced costs or increased benefits, while the pessimistic scenario considers unfavorable conditions, such as higher costs or lower benefits, providing a range of possible outcomes to assess the project’s resilience and feasibility. By highlighting the parameters that have the most significant effect on scenario outcomes, the sensitivity index enables stakeholders to focus on managing and mitigating the risks associated with these factors, ensuring a more resilient and well-informed decision-making process. The sensitivity index can be calculated using Equation (6) [107,108,109].

(6) $$Sensitivity\;index=\frac{{\mathrm{NPV}}_{opt}-{\mathrm{NPV}}_{pes}}{2}$$

**Figure 9:**
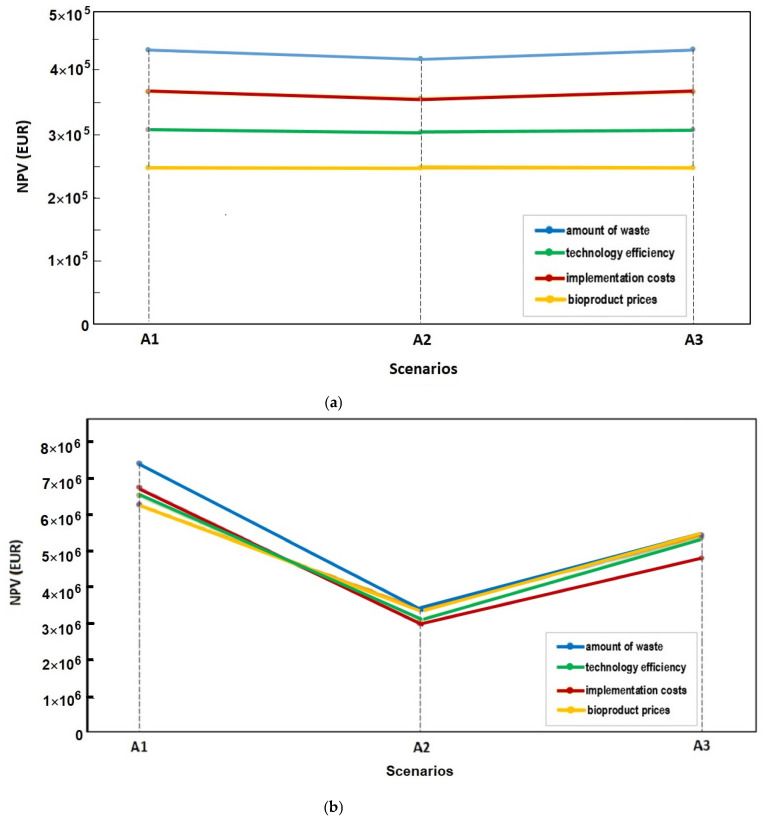
NPV values of alternatives A1–A3 under different scenarios: (**a**) Pessimistic scenario, depicting reduced net present values due to adverse conditions, and (**b**) Optimistic scenario, highlighting increased financial viability and potential profitability under favorable circumstances.

**Figure 10:**
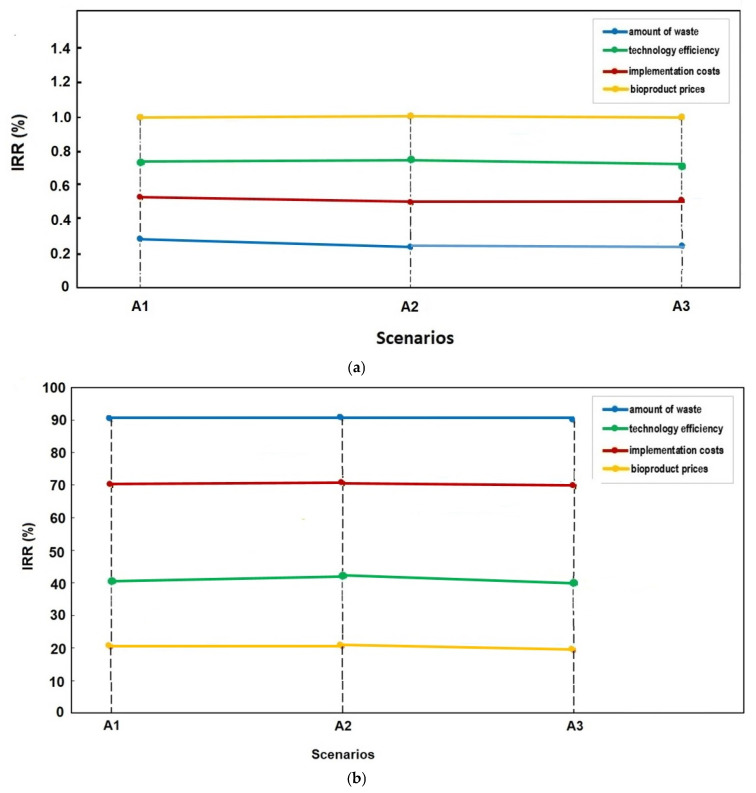
IRR values of alternatives A1–A3 under different scenarios: (**a**) Pessimistic scenario, reflecting lower potential returns and higher uncertainty, and (**b**) Optimistic scenario, showcasing favorable conditions with enhanced financial performance for each alternative.

The sensitivity analysis results for alternatives A1, A2, and A3, as illustrated in Figure 9 and Figure 10, offer critical insights into their financial performance and risk profiles under varying conditions.

In the pessimistic scenario (Figure 9a), all alternatives exhibit reduced NPVs, reflecting the adverse impact of increased costs or unfavorable market conditions. A1 maintains the highest NPV, followed by A2 and A3. The implementation costs for A1are the highest, followed by A3 and A2, indicating that A1’s profitability is more dependent on stable market conditions. The technology efficiency values also highlight A1’s leading position, with A3 slightly surpassing A2. Bioproduct prices follow a similar trend, with A3 having the highest value, making it a competitive option despite its higher sensitivity to cost fluctuations. In the optimistic scenario (Figure 9b), A1 remains the most profitable alternative, followed by A3 and A2. The implementation costs are again highest for A1, followed by A3 and A2 having significantly lower expenses. The technology efficiency values also suggest a strong financial case for A1, while A3 follows closely. Bioproduct prices further reinforce A1’s leading position with A3 performing better than A2.

The IRR analysis provides further insights into the financial attractiveness of each scenario. In the pessimistic scenario, A2 demonstrates the highest IRR for technology efficiency, followed by A1 and A3. However, in terms of bioproduct prices, A2 maintains the highest IRR, while A3 slightly outperforms A1. The variation in IRR values suggests that A2 is the most resilient alternative under unfavorable conditions, ensuring stability despite lower absolute profitability. In the optimistic scenario (Figure 10b), A1 achieves the highest IRR, followed closely by A2 and A3 in terms of waste utilization. However, A2 leads in implementation costs, while A1 and A3 remain slightly lower. The technology efficiency values suggest A2 performs better than A1 and A3. In terms of bioproduct prices, A2 again leads, while A1 and A3 show slightly lower returns.

The results confirm that A1 remains the most financially viable option, achieving the highest NPV and IRR values across both scenarios. However, its profitability is accompanied by increased sensitivity to market conditions, requiring careful risk management. A3 demonstrates significant potential, particularly in the optimistic scenario, where its financial returns improve relative to A2. This suggests that A3 is a viable alternative when market conditions are favorable. A2 consistently presents a more balanced and stable profile, with lower volatility across scenarios. Although it does not achieve the highest returns, its moderate NPV and IRR values position it as the safest choice for risk-averse investors seeking long-term financial predictability.

Figure 9 and Figure 10 collectively emphasize the trade-off between maximizing returns and ensuring financial stability. A1 is best suited for risk-tolerant stakeholders looking for high profitability, while A2 provides reliability with lower financial risks. A3, while not as stable as A2, offers a middle-ground option that balances risk and profitability, making it a competitive choice under favorable conditions. This analysis highlights the importance of scenario-based evaluation in determining the most appropriate food waste valorization strategy based on financial risk and expected returns.

**Figure 11:**
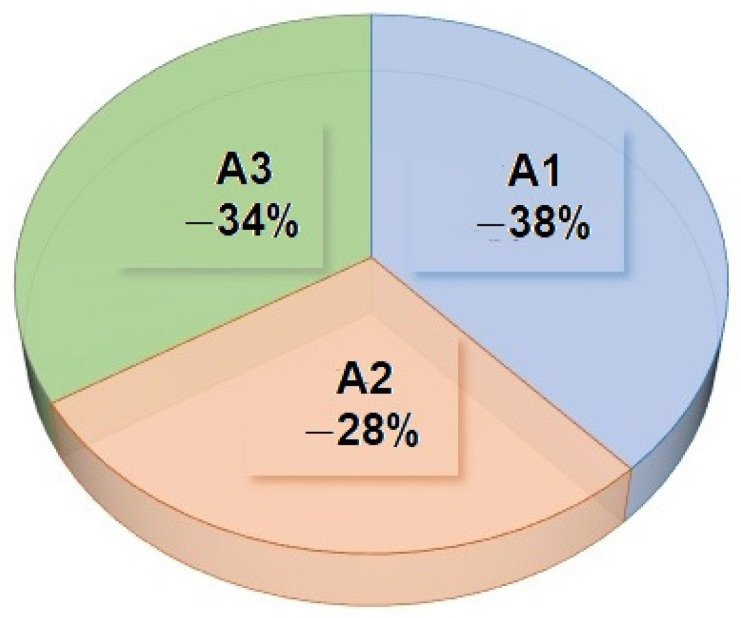
Sensitivity index values of the alternatives A1–A3, illustrating the varying degrees of financial and operational risk associated with each option, highlighting A1’s high sensitivity, A2’s resilience, and A3’s balanced adaptability in response to external fluctuations.

The sensitivity index analysis provides a comprehensive understanding of the varying risk profiles and operational suitability of the three alternatives, offering critical insights for stakeholders aiming to align their decisions with strategic objectives and risk tolerance (Figure 11).

A1 is characterized by its pronounced sensitivity to external factors, where even minor fluctuations result in significant financial deviations. This high-risk profile introduces a substantial level of uncertainty, making A1 less favorable for risk-averse stakeholders or projects that demand financial predictability. However, in environments where potentially high rewards justify elevated risks, A1 may still hold value for investors willing to adopt aggressive risk management strategies. This alternative necessitates robust contingency planning and the capacity to navigate volatile conditions, emphasizing its suitability for bold, high-stakes initiatives rather than stable, long-term investments.

A2 stands out as the most robust and stable option, showing negligible sensitivity to external changes. Its resilience to varying conditions underscores its suitability for projects that prioritize risk mitigation and consistent performance. A2 is an optimal choice for conservative investors or scenarios that demand financial and operational predictability, such as infrastructure development or public-private partnerships. The low-risk nature of A2 ensures dependable returns and reduces the likelihood of adverse impacts from unforeseen variables, making it a strategic choice for long-term ventures that focus on sustainability and steady growth.

A3 presents a moderate sensitivity to external changes, positioning itself as a middle-ground option between high-risk A1 and resilient A2. While not as stable as A2, A3 offers adaptability that suits dynamic and evolving environments. This alternative is particularly relevant for projects that require a combination of flexibility and measured risk exposure such as technology development or market-driven initiatives. A3’s balance makes it appealing to stakeholders seeking to manage risk without completely foregoing adaptability, ensuring sufficient responsiveness to shifting conditions while maintaining a degree of stability.

The sensitivity analysis highlights the strategic trade-offs between risk, adaptability, and performance consistency. A1, with its high sensitivity, caters to high-risk, high-reward strategies but requires significant preparedness for uncertainty. A2, as the most stable and predictable alternative, aligns with conservative and risk-averse objectives, ensuring reliability in performance. A3, occupying an intermediate position, provides flexibility with controlled risk, making it a versatile option in uncertain but manageable contexts.

Stakeholders should carefully evaluate these alternatives according to project goals, operational priorities, and risk appetites. This nuanced understanding enables informed decision-making, ensuring that the chosen alternative aligns with both immediate objectives and long-term strategic plans. Moreover, insights from the sensitivity index emphasize the importance of aligning project selection with anticipated market dynamics, resource availability, and financial resilience.

These results underscore the need for a dual focus on both profitability and risk when selecting a food waste valorization strategy. While high returns are desirable, the associated risks must also be considered to ensure the long-term viability of the chosen approach. A2’s ability to deliver steady economic and environmental outcomes across various scenarios makes it a robust choice for sustainable investment, while A1 and A3 require careful consideration of their respective risk levels before implementation. Ultimately, this analysis highlights the importance of selecting a strategy that not only maximizes returns but also ensures resilience in the face of fluctuating external factors.

## 7. Conclusions

This study provides an extensive assessment of enzymatic hydrolysis as a sustainable method for food waste valorization, highlighting its potential to generate bioethanol, bioactive peptides, and organic acids. Through a structured cost-benefit analysis, the results demonstrate that enzymatic hydrolysis provides significant economic, environmental, and social opportunities. From an economic point of view, the production of bioactive peptides has been shown to be the most viable option due to its high cost-benefit efficiency, while the production of organic acids shows the lowest economic feasibility, which needs further optimization. From an environmental perspective, enzymatic hydrolysis reduces landfill contributions and greenhouse gas emissions, thereby supporting resource circularity and sustainability objectives. From a social point of view, the process fosters job creation, enhances energy security, and transforms waste management into value-generating operations, contributing to the well-being of society at large.

This study, although promising, highlights some limitations and future directions. The economic viability of bioethanol and organic acid production remains sensitive to operational and market fluctuations, emphasizing the need for specific risk management strategies. Future research should focus on refining enzymatic processes, reducing costs, and exploring innovative applications for derived products to increase competitiveness. In addition, the scale-up of enzymatic hydrolysis technologies would need continued investment and policy support to maximize their economic, environmental, and social benefits. These efforts will be essential to position enzymatic hydrolysis as a foundation for circular economy initiatives and sustainable waste management practices.

## Figures and Tables

**Table 2:** Outline of practices and technologies to manage and reduce food waste.

Category	Solutions/Technologies	Description
Prevention and source reduction		
Smart inventory management	AI-based forecasting, real-time monitoring, freshness-tracking barcodes	Optimizes stock levels and prevents over-purchasing
Innovative packaging	Active, intelligent, and biodegradable packaging	Extends shelf life and indicates spoilage
Portion control tools	Portioning devices, menu engineering	Reduces over-serving in hospitality and catering
Redistribution and donation		
Food rescue platforms	Apps like Too Good to Go, OLIO, FoodCloud	Connects surplus food from businesses to consumers or charities
Real-time tracking	Blockchain systems	Ensures traceability and compliance in food donation
Policy incentives	Liability protection, tax benefits	Encourages food donations by reducing risks and offering financial incentives
Recycling and valorization		
Composting technologies	On-site composters, aerobic digesters	Converts food waste into fertilizer
Bioenergy production	Anaerobic digestion, pyrolysis, gasification	Produces biogas, biochar, or bio-oils from food waste
Insect bioconversion	Black Soldier Fly larvae (BSFL) systems	Converts waste into protein-rich feed and fertilizer
Upcycling and product development		
Upcycled food products	Spent grain-based snacks, fruit peel wraps	Transforms food waste into new consumable products
Biorefineries	Extraction of antioxidants, enzymes, and bio-pigments	Converts waste into high-value compounds
3D food printing	Reusing byproducts for printed food items	Creates new, edible items from food waste
Data-driven insights and AI		
IoT-enabled smart bins	Sensors in bins measuring waste volume and type	Provides actionable data for reduction strategies
Predictive analytics	AI models analyzing consumer behavior	Optimizes supply chain management and prevents overproduction
Education and awareness		
Consumer education	Awareness campaigns, apps, workshops	Promotes better storage, planning, and creative use of leftovers
Business training	Staff training, certification programs	Encourages waste reduction practices in retail and hospitality sectors
Policy and infrastructure support		
Date label standardization	Simplified “use by” vs. “best before” labeling	Prevents premature disposal of safe food
Waste reporting	Mandatory tracking of food waste	Helps monitor and reduce waste volumes
Centralized composting	Large-scale facilities for waste processing	Produces soil enhancers and supports agricultural sustainability
Cold chain improvements	Advanced refrigeration and transport systems	Reduces spoilage during distribution
Circular economy initiatives		
Community kitchens	Repurposing surplus food into meals for underserved communities	Supports food security and waste reduction
Zero-waste markets	Bulk purchases, reusable packaging	Minimizes packaging waste and encourages sustainable consumer habits

**Table 3:** Pathways for transforming food waste into valuable products.

Product Type	Source Materials	Processing Methods	Applications
Bioethanol	Carbohydrate-rich food waste (e.g., starchy residues, fruit peels, bread waste)	Fermentation (using yeast or bacteria)	Renewable energy (transport fuels, energy generation), reducing reliance on fossil fuels
Bioactive peptides	Protein-rich food waste (e.g., dairy byproducts, meat processing residues)	Enzymatic hydrolysis	Functional foods, nutraceuticals, dietary supplements with health benefits (antioxidant, antihypertensive)
Organic acids	Diverse organic waste (e.g., vegetable residues, fruit waste)	Fermentation (microbial processes)	Food preservation, pharmaceuticals, biodegradable plastics (e.g., polylactic acid from lactic acid)
Biofertilizers	Organic waste (e.g., food scraps, plant residues)	Composting, anaerobic digestion	Soil enrichment, sustainable agriculture
Animal feed	Protein and energy-rich food waste (e.g., bakery waste, fruit pulp)	Processing (drying, bioconversion with insects)	Livestock feed, aquaculture feed, reducing dependence on conventional feedstocks
Bioplastics	Starch and sugar-rich waste (e.g., corn waste, potato peels)	Fermentation, polymerization	Sustainable packaging, biodegradable plastic products
Enzymes	Food industry byproducts (e.g., fruit peels, dairy whey)	Fermentation, enzyme extraction	Industrial processes (textile, detergent, food industries)
Essential oils	Aromatic waste (e.g., citrus peels, herb trimmings)	Steam distillation, solvent extraction	Food flavoring, cosmetics, aromatherapy
Pigments	Colorful waste (e.g., beetroot pulp, carrot peels)	Solvent extraction, microbial synthesis	Natural colorants for food, textiles, and cosmetics
Biohydrogen	Food waste high in organic matter	Dark fermentation, photoermentation	Clean energy source for fuel cells and power generation
Biogas	Organic waste (e.g., food scraps, kitchen waste)	Anaerobic digestion	Renewable energy for heating, electricity, and vehicle fuel

**Table 4:** Overview of methodologies for evaluating the sustainability of food waste management systems.

Methodology	Key Indicators	Applications	Benefits
Life Cycle Assessment (LCA)	Greenhouse gas emissions, water consumption, energy usage, and land use.	Comparing the environmental performance of landfilling, incineration, composting, anaerobic digestion, and other waste management approaches.	Provides a detailed understanding of the environmental trade-offs and hotspots in waste management systems.
Cost-Benefit Analysis (CBA)	Net present value (NPV), benefit-cost ratio (BCR), and payback period.	Assessing the financial viability of implementing recycling programs, energy recovery facilities, and composting initiatives.	Helps decision makers prioritize cost-effective and socially beneficial solutions.
Material Flow Analysis (MFA)	Resource recovery rates, waste diversion percentages, and material efficiency.	Evaluating the efficiency of resource recovery strategies like composting and bioenergy production.	Identifies inefficiencies and potential points for intervention within the system.
Sustainability Assessment Frameworks	- Environmental: Emissions, energy use, water use, and biodiversity impacts. - Social: Public health, stakeholder engagement, and equity considerations. - Economic: Costs, job creation, and market impacts.	Policy evaluation, strategy comparison, and monitoring sustainability goals.	Multi-Criteria Decision Analysis (MCDA) for integrating diverse indicators into decision-making processes.
Circular Economy Metrics	Recycling rates, resource utilization efficiency, and the proportion of waste converted into value-added products.	Measuring the success of initiatives like composting, anaerobic digestion, and enzymatic hydrolysis.	Supports the transition toward closed-loop systems that reduce waste and conserve resources.
Carbon and Water Footprint Analysis	Total greenhouse gas emissions and water consumption per unit of food waste processed.	Identifying management practices with the lowest carbon and water footprints.	Highlights practices that contribute to climate change mitigation and water conservation.
Social Impact Assessments (SIA)	Public health outcomes, employment rates, and social acceptance of waste management technologies.	Assessing the societal benefits of food redistribution programs and community composting initiatives.	Ensures that waste management systems align with social equity and well-being goals.
Policy and Governance Analysis	Policy implementation success rates, stakeholder participation, and compliance levels.	Monitoring the impacts of national laws, such as food waste reduction targets, and international agreements like the Sustainable Development Goals (SDGs).	Identifies gaps in regulatory frameworks and areas for improvement in governance structures.

## Data Availability

The original contributions presented in the study are included in the article, further inquiries can be directed to the corresponding authors.
